# Mucoadhesive Hybrid System of Silk Fibroin Nanoparticles and Thermosensitive In Situ Hydrogel for Amphotericin B Delivery: A Potential Option for Fungal Keratitis Treatment

**DOI:** 10.3390/polym16010148

**Published:** 2024-01-03

**Authors:** Pratthana Chomchalao, Nuttawut Saelim, Supaporn Lamlertthon, Premnapa Sisopa, Waree Tiyaboonchai

**Affiliations:** 1 Department of Pharmaceutical Technology, Faculty of Pharmaceutical Sciences, Naresuan University, Phitsanulok 65000, Thailand; pratthana.c@ubu.ac.th; 2 College of Medicine and Public Health, Ubon Ratchathani University, Ubon Ratchathani 34190, Thailand; 3 Department of Pharmacy Practice, Faculty of Pharmaceutical Sciences, Naresuan University, Phitsanulok 65000, Thailand; 4 Centre of Excellence in Fungal Research, Department of Microbiology and Parasitology, Faculty of Medical Science, Naresuan University, Phitsanulok 65000, Thailand; 5 Department of Health and Cosmetic Product Development, Faculty of Food and Agricultural Technology, Pibulsongkram Rajabhat University, Phitsanulok 65000, Thailand

**Keywords:** mucoadhesive eye drops, amphotericin B, silk fibroin nanoparticles, composite formulations, ocular drug delivery

## Abstract

The purpose of this work was to investigate the feasibility of a novel ophthalmic formulation of amphotericin B-encapsulated silk fibroin nanoparticles incorporated in situ hydrogel (AmB-FNPs ISG) for fungal keratitis (FK) treatment. AmB-FNPs ISG composites were successfully developed and have shown optimized physicochemical properties for ocular drug delivery. Antifungal effects against *Candida albicans* and in vitro ocular irritation using corneal epithelial cells were performed to evaluate the efficacy and safety of the composite formulations. The combined system of AmB-FNPs-ISG exhibited effective antifungal activity and showed significantly less toxicity to HCE cells than commercial AmB. In vitro and ex vivo mucoadhesive tests demonstrated that the combination of silk fibroin nanoparticles with in situ hydrogels could enhance the adhesion ability of the particles on the ocular surface for more than 6 h, which would increase the ocular retention time of AmB and reduce the frequency of administration during the treatment. In addition, AmB-FNP-PEG ISG showed good physical and chemical stability under storage condition for 90 days. These findings indicate that AmB-FNP-PEG ISG has a great potential and be used in mucoadhesive AmB eye drops for FK treatment.

## 1. Introduction

The incidence of corneal infection by fungi or fungal keratitis (FK) has increased worldwide in recent years and can cause blindness [1]. Several risk factors, such as corneal injury, extended wear of contact lenses, ocular surface diseases, and topical steroid use have been associated with the development of FK [2]. In tropical regions, filamentous fungi, such as *Fusarium* and *Aspergillus* species, are predominant causes of FK, whereas yeasts, such as *Candida* species, are a less frequent cause [3]. However, corneal infections caused by *Candida* spp. are complicated to treat due to their ability to form a biofilm that leads to antifungal resistance [4]. Amphotericin B (AmB) is an effective drug in the treatment of FK because it has broad spectrum activity against most fungi, especially *Candida* spp. with a low incidence of clinical resistance [5]. Unfortunately, the therapeutic use of AmB is limited by its toxicity and poor solubility, which make it difficult to fabricate an optimum ophthalmic formulation. Furthermore, AmB eye drops are not commercially available, and the conventional intravenous dosage form of AmB deoxycholate (Fungizone^®^, Bristol-Myers Squibb, Montreal, QC, Canada) is mostly off-label and used for FK treatment [6,7]. The main problem of this extemporaneous preparation is eye irritation from the sodium deoxycholate in the formulation, leading to poor patient compliance [8]. Less toxic lipid-based formulations of AmB have been developed, but these forms are very expensive, have low stability in aqueous solution, and are unavailable in developing countries, thus restricting their use [9]. In addition, AmB delivered through conventional eye drops is rapidly eliminated from the ocular surface due to the protective mechanisms of the eye, such as reflex blinking and tear dilution, and entry into the systemic circulation via nasolacrimal drainage [10]. High doses and high-frequency application have been used to achieve therapeutic efficacy of treatment of the fungal infection, which can result in serious systemic side effects [11,12,13]. Accordingly, many studies have focused on developing AmB ophthalmic formulations with high effectiveness, low toxicity, and good stability using drug delivery systems such as polymeric nanoparticles [14,15], nanostructure lipid carriers [16,17], liposomes [18], microneedles [19], and nanofiber with in situ gelling [20]. Nevertheless, no licensed AmB topical ophthalmic formulations are available.

Consequently, our research focuses on the use of combination systems of nanoparticles and thermosensitive in situ hydrogels to fabricate a promising ophthalmic AmB. Nanoparticles offer various outstanding advantages for ocular drug delivery including improved bioavailability of the drugs and extended drug retention time, enabling targeted delivery and reduced side effects [21]. Several studies reported that small nanosized particles, ranging from 50 to 400 nm, could overcome the ocular barrier, enhancing bioavailability of poorly soluble drugs and prolonging the contact time of the drugs on the eye [22,23]. Furthermore, nanoparticles shield the drug from interacting directly with normal cells, thus minimizing its side effects. As is known, AmB activity and toxicity depend on the aggregation state of AmB, which is influenced by carriers. The incorporation of AmB into nanoparticles having a monomeric form has been shown to enhance antifungal activity and reduce the irritation induced by AmB [24]. However, low viscosity nanodispersion can be quickly cleared from the eye by tears or blinking, resulting in inadequate drug retention on the ocular surface. To overcome this drawback, combining the nanoparticles with the in situ gelling system can be an effective approach to increase viscosity upon application. This system has the capability to undergo a phase transition in response to environmental triggers such as temperature, pH, or ions [25]. The liquid form of in situ hydrogel can be easily administered as eye drops and it can transform into a gel after contact with the ocular surface, thereby increasing the drug’s residence time, and decreasing the frequency of administration and dosing [12,26].

Based on our previous studies, we successfully developed a ready-to-use AmB ophthalmic formulation by formulating a combined system of AmB-encapsulated silk fibroin nanoparticles incorporated in thermosensitive in situ hydrogel (AmB-FNPs ISG) [27] to enhance ocular bioavailability and increase precorneal residence time of AmB. To create the thermosensitive in situ gelling system, Pluronic F127-based formulations were utilized in this work. Pluronic F127 is a thermoresponsive polymer widely employed in pharmaceutical formulations. At low temperature, Pluronic exists in a liquid state and can self-assemble to form small micelles due to their amphiphilic structure. With a temperature increase, the micellar structures pack closely together to form a three-dimensional network, leading to the formation of the gel [28]. AmB-FNPs ISG possesses a liquid form at ambient temperature and rapidly converts to a gel at ocular temperature, offering ease of administration while prolonging its retention on the eye. These novel formulations present pale yellowish solutions, high transparency, and optimum pH and osmolality. All AmB-FNPs incorporated into the thermosensitive in situ hydrogel showed high entrapment efficiency with a mean particle size of ~200 nm, which could enhance AmB bioavailability and cause no ocular irritation. Moreover, the results from FTIR, XRD, and molecular aggregation studies revealed that highly hydrophobic AmB was encapsulated in FNPs in an amorphous form, which could reduce the aggregated toxicity of AmB. These findings demonstrated that AmB-FNPs ISG formulations possess great potential physicochemical properties for topical ocular application. Accordingly, the present study aims to demonstrate the antifungal activity, mucoadhesive properties, reduced ocular irritation, and stability of the AmB-FNPs ISG for the treatment of FK.

## 2. Materials and Methods

### 2.1. Materials

Amphotericin B, as the active pharmaceutical ingredient, was bought from Bio Basic Inc. (Toronto, ON, Canada). Intravenous amphotericin B was supplied by Biolab (Samutprakarn, Thailand). Polyethylene glycol 400 (PEG) was purchased from Nam Siang Co. Ltd. (Bangkok, Thailand). Branched polyethylenimine (PEI), hyaluronic acid, mucin from porcine stomach type II, hydrocortisone, insulin from bovine pancreas, and fluorescein isothiocyanate were ordered from Sigma-Aldrich (St. Louis, MO, USA). Poloxamer 407 (Pluronic^®^ F127) was acquired from BASF (Florham Park, NJ, USA). Muller-Hinton agar and Sabouraud Dextrose agar were purchased from HIMEDIA (Mumbai, India). Roswell Park Memorial Institute (RPMI) 1640 medium, keratinocyte serum free medium (K-SFM), bovine pituitary extract (BPE), epithelial growth factor (EGF), and Penicillin/ Streptomycin solution were ordered from Gibco (New York, NY, USA). Thiazolyl blue tetrazolium bromide was acquired from Amresco^®^ (Solon, OH, USA). Crystal violet was supplied by Riedel-de Haën (Munich, Germany). All other reagents were of analytical grade or higher.

### 2.2. Preparation and Characterization of AmB-FNPs ISG Composites

The soluble silk fibroin (SF) was extracted as reported before by dissolving small fibers of degummed silk yarn (Bodin Thai Silk Khorat Co., Ltd., Nakhon Ratchasima, Thailand) in a mixture of CaCl_2_:H_2_O:Ca(NO_3_)_2_:EtOH (30:45:5:20 weight ratio) solvent at 80 °C for 4 h [29]. Then, pure SF solution was obtained after dialysis in distilled water for 3–5 days using snakeskin dialysis tubing (10,000 Da MWCO). Three different amphotericin B-encapsulated silk fibroin nanoparticles (AmB-FNPs)—uncoated AmB-FNP (AmB-FNP), AmB-FNP crosslinked with PEI (AmB-FNP-PEI), and AmB-FNP coated with PEG 400 (AmB-FNP-PEG) were prepared following our previous study using the desolvation method [27]. To enhance the entrapment efficiency of AmB and promote the interaction between AmB-loaded nanoparticles and mucin at the ocular surface, cationic polymer PEI and a mucoadhesive polymer PEG 400 were utilized for modification of the surface of silk fibroin nanoparticles. Briefly, an aqueous 1% *w*/*v* SF solution (2% *w*/*v* SF was used instead of 1% *w*/*v* SF for AmB-FNP-PEI) was injected dropwise into mild stirred absolute ethanol, ethanol/1% *w*/*v* PEI solution (pH 7.0), or ethanol/1% *w*/*v* PEG 400 solution with AmB (15 mg per 30 mL). The SF:Ethanol ratio tested was 10: 20 *v*/*v*. The spontaneously formed particles were centrifuged at 12,000 rpm for 60 min, washed thrice with DI water, and sonicated at 40% amplitude for 60 s. Finally, all AmB-FNPs were lyophilized and kept in the refrigerator for further experiment. 

Mean particle size, polydispersity index (PDI), and zeta potential of all AmB-FNPs were measured at 25 °C using a Zetasizer Ultra (Malvern Panalytical Ltd., Malvern, UK) by diluting the samples in DI water and examining them in triplicate. In addition, entrapment efficiency (EE) and drug loading capacity (DL) were evaluated using an indirect method. After centrifugation, the amount of unentrapped AmB in the supernatant was analyzed using a UV–Visible spectrophotometer (Genesys 10 s, Thermo Scientific, Waltham, MA, USA) at 405 nm to calculate %EE and %DL. 

To determine the crystallinity of AmB in the FNPs, X-ray diffractometry (XRD) analysis was performed. Pure AmB powder, SF, freeze-dried powder of AmB-FNPs, blank FNPs, and physical mixes of AmB and blank FNPs were examined with an X-ray diffractometer (D2 Phaser, Bruker AXS Inc., Madison, WI, USA) at 45 kV and 36 mA with a scan speed of 2°/min and scanning from 10–30°.

The prepared AmB-FNPs were incorporated in 2 optimal in situ hydrogel bases, namely, 19% *w*/*v* Pluronic^®^ F127 (F127) and 18% *w*/*v* Pluronic^®^ F127 blended with 0.2% *w*/*v* hyaluronic acid (F127/HA). Briefly, AmB-FNP dispersion and the in situ hydrogel solution were separately prepared using the cold method. Lyophilized AmB-FNPs were re-dispersed in deionized (DI) water and sonicated at 40% amp for 60 s. The F127 and F127/HA solution were prepared by dispersing F127 powder in cold DI water and hyaluronic acid (HA) aqueous solution, respectively. Then, both polymer dispersions were kept in a refrigerator for at least 24 h for completely dissolution. The AmB-FNP dispersions were mixed into an equal volume of F127 or F127/HA solution at 4 °C by constant stirring until homogenous, and the final dose of AmB was equivalent to 150 μg/mL. All AmB-FNPs ISG formulations were kept in a refrigerator and protected from light for further investigation. The physical properties of AmB-FNPs ISG formulations were characterized in term of clarity, gelling capacity, pH, osmolality, optical transmittance, viscosity, rheological behavior, and sol–gel transition temperature.

Visual examination against black and white backgrounds was used to determine the clarity of the composite formulations.

The gelling capacity was performed by dropping 30 μL of each composite formulation into a test tube containing 2 mL of stimulated tear fluid (STF) at pH 7.4 equilibrated at 35 ± 1 °C. The appearance of gel forming was visually evaluated. 

The pH and osmolality of the composite formulation were measured using a pH meter (SK20, Mettler-Toledo, Zurich, Switzerland) and freezing point depression osmometer (Osmomat^®^ 030, Gonotec, Berlin, Germany), respectively. All measurements were made in triplicate and the data are reported as a mean ± SD.

The optical transmittance was performed on a UV–Visible spectrophotometer. Fifty microliters of each composite formulation was smeared on the outside surface of a quartz cuvette and maintained at 35 ± 1 °C. Then, the % transmittance was measured under the visible wavelength from 381 to 780 nm and the empty cuvette was used as a blank. All tests were conducted in triplicate and the data are expressed as mean ± SD. 

The viscosity and rheological behavior of AmB-FNPs ISG formulations were investigated using a cone and plate viscometer (DV3T model, Brookfield, MA, USA). The viscosity of each formulation was measured at 25 ± 1 °C (room temperature) or 35 ± 1 °C (ocular surface temperature) with a constant shear rate at 20 s^−1^. The rheology tests were conducted at 35 ± 1 °C with increasing shear rate from 1 to 80 s^−1^ and the rheograms were plotted between the viscosity and shear rate. The sol–gel transition temperatures of both AmB-FNPs-F127 and AmB-FNPs-F127/HA were measured at a 10 rpm spindle speed using a Brookfield rheometer (RST-CVS-PA, Brookfield, USA) with increasing temperatures from 20 °C to 40 °C controlled by a Peltier plate. The data were plotted as viscosity versus temperature and the gelling temperature was defined as the temperature when the rigid gel state formed. 

### 2.3. Stability Study

A stability study was performed to determine the physicochemical stability of the formulations under storage conditions. AmB-FNP-PEG F127 ISG and AmB-FNP-PEG F127/HA ISG were represented as a candidate ophthalmic formulation for the stability study. Both formulations were stored at 4 ± 1 °C (refrigerated temperature) in darkness for a period of 3 months and evaluated at intervals of 7, 14, 21, 30, 60, and 90 days for clarity, pH, gelling capacity, and drug content. 

### 2.4. In Vitro Antifungal Activity

#### 2.4.1. Screening of the Antifungal Activity

The antifungal effects of the AmB-FNPs dispersion and AmB-FNPs-ISG were tested against a standard strain of *Candida albicans* (TISTR 5779). The agar well diffusion technique was used to screen the antifungal effects of all formulations [30]. Briefly, a *C. albicans* suspension (1 × 10^6^ CFU/mL) was prepared in 0.85% NaCl and swabbed evenly on the surface of Muller–Hinton agar containing 2% glucose and methylene blue. Then, 10 μg/mL of AmB stock solution of standard AmB, AmB deoxycholate, AmB-FNPs, and AmB-FNPs-ISG was prepared in sterile water. Subsequently, holes having a diameter of 6 mm were made in the inoculated agar plates and filled with 20 μL of stock solution of the samples. In addition, sterile water and plain in situ hydrogels were also added to the wells as a negative control. The samples were then incubated for 24 h at 37 °C. Antifungal effects were determined as the absence of fungal growth in the area surrounding the hole and the diameter of inhibition zone was measured using a Vernier caliper. These analyses were performed in triplicate.

#### 2.4.2. Minimum Inhibitory and Minimum Fungicidal Concentration Test

The minimal inhibitory concentration (MIC) was evaluated using the broth dilution technique according to the EUCAST guidelines [31]. The stock solution of standard AmB powder was dissolved in DMSO while AmB deoxycholate, AmB-FNPs, and AmB-FNPs-ISG were prepared in DI water. All samples were two-fold serial diluted in a 96-well plate with RPMI 1640 medium, 2% glucose, and 25 mM HEPEs, pH 7.0, and with the AmB concentration ranging from 0.0312 to 16 μg/mL. The *C. albicans* suspension was prepared in RPMI 1640 medium and further inoculated in each well of the 96-well plates to obtain a final inoculum concentration of ~1–5 × 10^5^ CFU/mL. Consequently, the final AmB concentration ranged from 0.0156 to 8 μg/mL. A *Candida* suspension cultured in the media without the samples served as a positive control, and the mixture of media and the samples without the *Candida* suspension served as a negative control. The assay plates were used to measure the optical density at 530 nm using a microplate reader (Synergy H1 Hybrid Reader, BioTek, Agilent, CA, USA) prior to and after incubation at 37 °C, for 24 h. The MIC_90_, defined as the lowest test concentration that inhibited 90% of the fungal growth, was calculated for all formulations. 

After MIC determination, 10 μL aliquots of media from each well with no fungal growth were dropped on Sabouraud Dextrose Agar (SDA) plates and incubated at 37 °C for 24 h. Finally, the minimal fungicidal concentration (MFC) was defined as the lowest AmB concentration that showed no detectable growth on the SDA surfaces. All experiments were conducted in triplicate. 

### 2.5. In Vitro Mucoadhesive Study

All blank FNPs were covalently bound with fluorescent dye of fluorescein isothiocyanate (FITC) for both in vitro and ex vivo mucoadhesive studies. Briefly, 10 mg of lyophilized blank particles was re-dispersed in 1 mL of carbonate buffer pH 9. Then, 50 μL of 1 mg/mL FITC solution was slowly added into the dispersion with gentle and continuous stirring. The mixture was incubated in the dark for 24 h at 4 °C. The FITC conjugated with FNPs (FITC-FNPs) was then separated from unbound FITC by centrifugation at 16,000 rpm for 30 min and washed 2 times with DI water. The FITC-FNPs were freshly made before each experiment. 

The flowing liquid test was performed to investigate the mucoadhesive properties of the prepared formulations. This method involves washing the formulation with an appropriate artificial fluid at a constant flow rate while the residence time of the formulation is determined visually or fluorometrically [32]. The in vitro mucoadhesive test was conducted under a condition mimicking the pre-ocular surface. A mucus layer was prepared by soaking a hydrophilic membrane (polycarbonate membrane, 0.2 μm pore size, 6 mm diameter, Isopore^TM^, Merck, Germany) in 0.1% of aqueous mucin solution for 24 h. Then, 10 μL of the FITC solution, FITC-FNPs dispersion, and FITC-FNPs-ISG was applied as a single drop at the center of the membrane and incubated at 35 ± 1 °C for 2 min to induce a gel forming an in situ hydrogel formulation. The membrane was then immediately washed with a continuous flow of STF solution (pH 7.4, 35 ± 1 °C) at a flow rate of 10 μL/min controlled by a peristatic pump. Then, STF containing the eliminated FITC was collected at 5, 15, 30, 60, 120, 240, and 360 min, and the fluorescence intensity was measured using a microplate reader at excitation (Ex) and emission (Em) wavelengths of 495 nm and 525 nm, respectively. The amount of eliminated FITC in STF was calculated according to the standard curve of FITC (concentration range 0.25–20 ng/mL) and the percentage of FITC remaining on the mucus membrane was calculated as follows:

(1) $$\%FITC\;remaining=\frac{Initial\;amount\;of\;FITC-Amount\;of\;eliminated\;FITC\;at\;time\;point}{Initial\;amount\;of\;FITC}\times 100$$

### 2.6. Ex Vivo Mucoadhesive Study

The ex vivo mucoadhesive test using fresh porcine cornea and an apparatus setup modified from Chiyasan et al. [33] was performed. Porcine eyes were obtained from the local slaughterhouse, Phitsanulok, Thailand (license number PC 06 47001/2536), and kept in ice-cold PBS, pH 7.4, containing 1% *v*/*v* antibiotic solution until used (less than 8 h after death). Six millimeters of the corneal tissue was excised with a surgical blade and mounted onto a glass slide; 48 porcine corneas were obtained in this experiment. Ten microliters of the FITC solution, FITC-FNP-PEG dispersion, and FITC-FNP-PEG-ISG was dropped on the corneal surface. The samples were incubated at 35 °C for 5 min to ensure the gelation form of in situ hydrogel. Then, the tissue was placed in contact with a continuous stream of STF pH 7.4 at 35 ± 1 °C with a flow rate of 10 μL/min to mimic the eye blinking. At the time points of 30, 120, 240, and 360 min, cryostat sections of the cornea tissue were removed and imaged using fluorescence microscopy to visualize green fluorescence of FITC adhering to the tissue. 

### 2.7. In Vitro Irritation Study

#### 2.7.1. MTT Assay

The human corneal epithelial (HCE) cell line (ATCC CRL-11135) was used for cell viability investigation. HCE cells were cultured in the complete growth media of K-SFM supplemented with 0.05 mg/mL BPE, 5 ng/mL of EGF, 500 ng/mL hydrocortisone, 0.005 mg/mL insulin, and 1% *v*/*v* Penicillin/Streptomycin at 37 °C with 5% CO_2_. The influence of the prepared formulations on cell viability was investigated using an MTT assay, which was modified following the short time exposure (STE) protocol recommended for an alternative ocular irritation assay [34]. Briefly, HCE cells were trypsinized and seeded into a 96-well plate at 1 × 10^4^ cells/well and cultured in the complete growth media at 37 °C with 5%CO_2_ for 24 h. Then, the culture media was removed and the HCE cells were exposed to AmB-FNPs and AmB-FNPs-ISG formulations for comparison with the control. After 12 h, the medium containing the samples was discarded carefully, and each well was washed with sterile PBS, pH 7.4. The HCE cells were further incubated with MTT solution (final concentration of 0.5 mg/mL) at 37 °C in the dark for 2 h. Finally, the MTT solution was removed, and the formazan crystals were dissolved with dimethyl sulfoxide (DMSO). The absorbance of each well was read on a microplate reader at 595 nm. The percentage of cell viability was calculated in comparison to the vehicle treated cells.

#### 2.7.2. Crystal Violet Staining

The HCE cells were cultured and treated with the prepared formulations for 12 h, similar to the MTT assay. After the treatment times, the supernatant was discarded, and the cells were washed 3 times with PBS pH 7.4. The treated cells were fixed with 4% paraformaldehyde for 3 h at room temperature. After fixation, the HCE cells were stained with 0.5% *w*/*v* crystal violet solution and incubated for 30 min, then washed with tap water to remove excess staining. Cell samples were air dried and visualized under a light microscope.

### 2.8. Statistical Analysis

The mean ± SD (standard deviation) is presented for quantitative experiments. The statistical analysis was conducted using SPSS 17.0 software (Chicago, IL, USA). The significance was evaluated using one-way analysis of variance (1-way ANOVA) along with Tukey’s post hoc test, and *p* < 0.05 was considered as statistically significant. 

## 3. Results and Discussion

### 3.1. AmB-FNPs ISG Composites Characterization

From our previous study, we successfully prepared the novel formulation of AmB-FNPs-ISG. Their physicochemical properties are summarized in Table 1. All AmB-FNPs exhibited a mean particle size of ~200 nm with high entrapment efficiency of up to 63%. The aggregation study of AmB and XRD results from our previous study indicated that AmB was entrapped in the FNPs with an amorphous form [27]. The decrease in the crystallinity of AmB may be attributed to the interaction of AmB and silk fibroin via hydrophobic interaction. All prepared AmB-FNPs-ISG formulations possessed optimized osmolality, pH, and viscosity for ocular application. The rheological evaluation of all composite formulations exhibited pseudoplastic flow behavior after gel formation at 35 ± 1 °C, which allowed good spreadability on the ocular surface and ease of eye blinking. 

**Figure 1:**
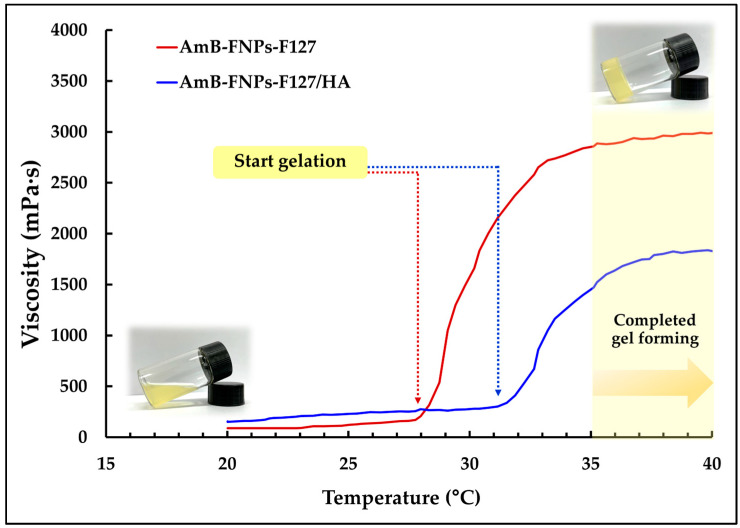
Average viscosity vs. temperature profiles of AmB-FNPs ISG.

In addition, they undergo temperature-dependent sol–gel transition, from a flowing solution at ambient temperature (25 ± 1 °C) to a non-flowing gel at ocular temperature (35 ± 1 °C), making them easy to administer while enhancing the retention time of the drug on the eye surface. According to the viscosity vs. temperature curve, it was observed that both types of in situ hydrogels, AmB-FNPs-F127 and AmB-FNPs-F127/HA ISG, exhibited a constant viscosity at low temperature and their viscosity significantly increased as the temperature increased, indicating the gelation process (Figure 1). Furthermore, AmB-FNPs-F127 ISG showed a lower sol–gel transition temperature (~28 °C) than AmB-FNPs-F127/HA ISG (~31 °C), indicating AmB-FNPs-F127 ISG underwent gelation faster than AmB-FNPs-F127/HA ISG. After completed gel formation, AmB-FNPs-F127 ISG revealed higher viscosity than AmB-FNPs-F127/HA ISG, suggesting higher gel strength. These results may be related to the higher Pluronic concentration in the AmB-FNPs-F127 ISG formulation. Pluronic molecules can promptly self-assemble to form micelles in the aqueous media due to their amphiphilic nature, and the number of micelles increased when Pluronic concentration increased. The increasing temperature results in packing of micelles to form a large micellar crosslinked network, leading to gel formation [35]. Hence, the number and size of Pluronic micelles in the AmB-FNPs-F127 ISG formulation increased, resulting in a higher number of crosslinked micelles, and then leading to faster gelation and greater viscosity. 

There is limited literature available on the development of composite systems involving nanoparticles and hydrogels for the ophthalmic delivery of AmB. Göttel et.al. developed AmB-loaded in situ gelling nanofiber to enhance the solubility of AmB for keratomycoses treatment [20]. However, the entrapment efficiency of AmB in PLGA nanoparticles for this study was ~40% and no mucoadhesive study was conducted. In addition, Elhabal et.al. recently examined a thermosensitive hydrogel of AmB and Lactoferrin combination-loaded PLGA-PEG-PEI nanoparticles for eradication of ocular fungal infections [36]. This study exhibited high entrapment efficiency of AmB > 90% and good stability, but the mucoadhesion of the formulation was not reported in this study. 

### 3.2. Stability Study

The stability of AmB in topical ophthalmic dosage form is a challenge for pharmaceutical research. As is known, the stability of AmB in aqueous solution under heat condition is very low; hence, AmB products are stored at a low temperature of ~2–8 °C. Curti et al. reported that conventional AmB eye drops are stable for fewer than 15 days under ambient temperature and for 60 days under refrigeration conditions (2–8 °C) [37]. Moreover, Chanell et al. investigated the stability of ready-to-use amphotericin B solubilized in 2-hydroxypropyl-γ-cyclodextrin (AB-HP-γ-CD) formulations. They found that their formulation showed AmB instability after 28 and 56 days at 25 °C and 5 °C, respectively [9].

**Figure 2:**
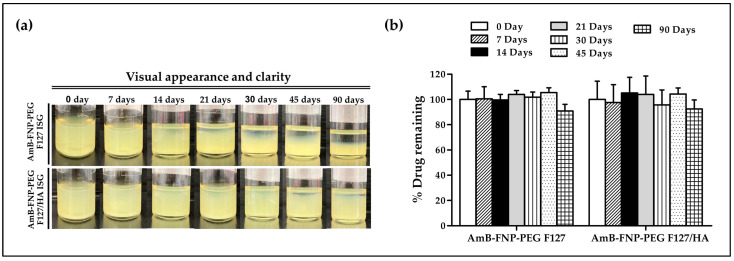
Stability of AmB-FNP-PEG-F127 ISG and AmB-FNP-PEG-F127/HA ISG under storage conditions (4 °C). (**a**) Visual appearance and (**b**) drug remaining of AmB in the formulations at different time points (mean ± SD, *n* = 3).

In this study, AmB-FNP-PEG-ISG was selected as a representative to study the stability of the composite formulations. Samples were kept at 4 °C for 90 days. At all predetermined time points, both AmB-FNP-PEG-ISG samples showed good gelling capacity with pH ~7. However, both formulations exhibited phase separation when the storage time was increased (Figure 2a). This result may be associated with agglomeration of the particles in the colloidal system. Interestingly, AmB-FNP-PEG-F127 ISG showed higher sedimentation of nanoparticles on the bottom of vial than AmB-FNP-PEG-F127/HA ISG. This result could be attribute to electrostatic stabilization because the negative charge of hyaluronic acid enhances the strong repulsion of the AmB-FNP-PEG particles in the F127/HA ISG network [38]. Although AmB-FNP-PEG-ISG showed a phase separation, all AmB-FNPs-ISG could transform to a homogeneous dispersion after mild shaking. Interestingly, Figure 2b shows no significant difference in the drug remaining of AmB-FNP-PEG-ISG between the beginning and end of the storage time, indicating good stability of AmB in the prepared formulation. This result may be attributed to the incorporation of AmB into silk fibroin nanoparticles, which could protect AmB degradation from the hydrolysis mechanism in the aqueous media, leading to increased stability of AmB.

### 3.3. Antifungal Efficacy

**Figure 3:**
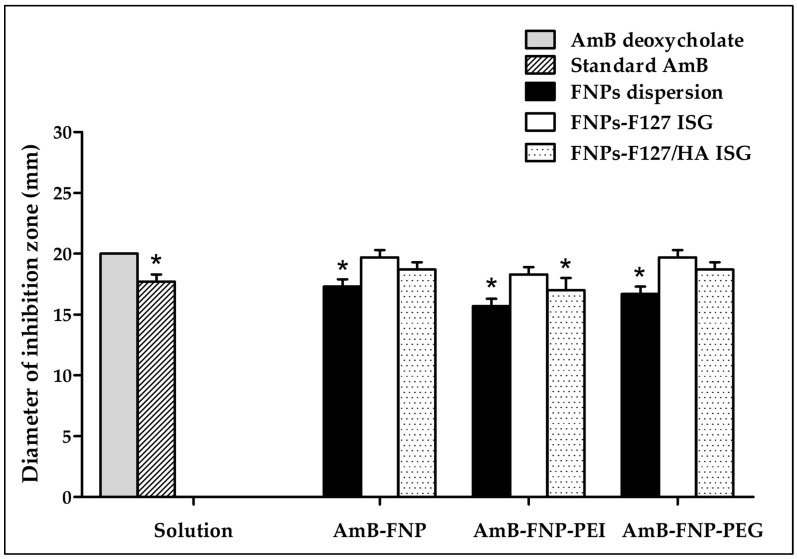
The antifungal activity of AmB deoxycholate, standard AmB, AmB-FNPs, and AmB-FNPs ISG against *C. albicans* according to the agar well diffusion technique. * Significant at *p* < 0.05 compared with AmB deoxycholate.

The potential of using the novel AmB-FNPs-ISG as topical ophthalmic formulation for fungal keratitis was investigated. The agar well diffusion technique was used to screen the antifungal effect of the prepared formulations compared with AmB deoxycholate, a commercial formulation. This study was conducted by measuring the inhibition zone of the sample against *C. albicans*. As expected, the inhibition zone was observed around the holes of AmB-FNPs, AmB-FNPs-ISG, and the positive controls (standard AmB and AmB deoxycholate), whereas the negative controls (sterile water, blank F127 ISG, and blank F127/HA ISG) showed no inhibition zone, indicating no antifungal activity (Figure 3). AmB deoxycholate showed the highest inhibition zone (~20 mm), which was significantly different from that of standard AmB (~17 mm), AmB-FNP dispersion (~17 mm), AmB-FNP-PEI (~16 mm), AmB-FNP-PEG dispersion (~17 mm), and AmB-FNP-PEI F127/HA (~17 mm). Interestingly, the inhibition zone of all AmB-FNPs-ISG formulations, with the exception of AmB-FNP-PEI F127/HA, was slightly greater than that of their FNP counterparts. These results may be associated with the effect of surfactants in the formulations, deoxycholate in the commercial AmB and Pluronic in the in situ hydrogels, which could enhance the membrane-associated target of AmB and increase the permeability of the fungal membrane [39]. Although the agar well diffusion technique is a widely used for assessing the antimicrobial activity of various drug formulations, the outcomes of this technique are variable due to inherent limitations in the hydrophilicity and viscosity of the formulations, as well as the interaction with the agar component [40,41]. To this end, we combined the agar diffusion test with MIC and MFC tests to confirm the potential of the prepared formulations for treatment of ocular infections. 

In addition, MIC_90_ and MFC of all formulations against *C. albicans* were investigated (Table 2). AmB deoxycholate and standard AmB showed similar MIC_90_ and MFC values of 0.0625 μg/mL and 0.5 μg/mL, respectively. However, AmB-FNPs exhibited higher MIC_90_ and MFC values of 0.25 μg/mL and 1 μg/mL, respectively. The lower antifungal activity could be explained by some of the AmB in FNP formulations being restricted in the particles due to strong interaction between AmB and silk fibroin, as observed from dissolution studies, which showed ~50% AmB release within 5 h [27].

Interestingly, AmB-FNPs-ISG formulations exhibited higher antifungal activity than AmB-FNPs with the MIC_90_ value of 0.125 μg/mL and MFC value of 0.5 μg/mL, close to those of AmB deoxycholate. This may be due to the support from Pluronic, a surfactant in the hydrogel matrix, which could enhance the diffusion of AmB across the fungal cells, leading to an increase in the fungal cell death. These results indicate that the prepared AmB-FNPs-ISG have potential antifungal activity similar to that of the marketed AmB deoxycholate.

### 3.4. In Vitro Mucoadhesive Study

**Figure 4:**
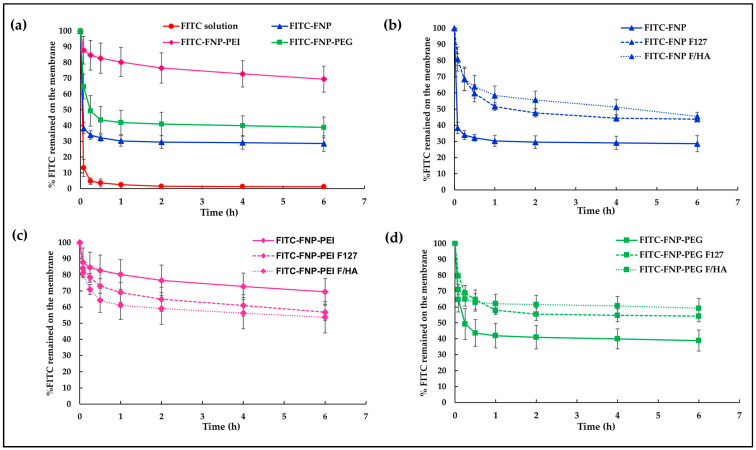
The in vitro mucoadhesive properties of FITC-labeled FNPs and FITC-FNP-ISG. (**a**) %FITC remaining on the mucus membrane of FITC solution compared with three types of FITC-FNP dispersion; (**b**) %FITC remaining of FITC-FNP dispersion; (**c**) %FITC remaining of FITC-FNP-PEI dispersion; and (**d**) %FITC remaining of FITC-FNP-PEG dispersion compared with their in situ hydrogel composites.

To track the mucoadhesion of particles on the mucus layer, all FNPs were labeled with FITC, a green fluorescent dye. Generally, FITC is widely used to label proteins via the reaction of isothiocyanate groups of FITC and amine groups in the protein. Therefore, isothiocyanate groups of FITC could attach with residual amine groups of silk fibroin. In this study, FITC-labeled FNP, FNP-PEI, and FNP-PEG were prepared following the method of Pham et al. [42]. To examine the in vitro mucoadhesive properties of FITC-labeled FNPs and FITC-labeled FNP in situ hydrogels, the hydrophilic membrane soaked with mucin solution was used to mimic a mucus membrane. Figure 4 illustrates the percentage of FITC remaining on the mucus membrane after continuous flow of STF. After 5 min of fluid flow, the membrane instilled with FITC solution showed only ~13% remaining, while those instilled with FITC-FNP, FITC-FNP-PEG, and FITC-FNP-PEI showed 38%, 65%, and 88% remaining, respectively (Figure 4a). Moreover, nearly 100% loss occurred after 1 h of fluid flow when instilled with FITC solution, whereas all FITC-FNPs showed % FITC remaining of up to 30%. These results indicated that small particles of FNPs could enhance FITC adherence to the mucus membrane because the large surface area of the particles can increase the adhesion to the mucus membrane [43]. 

Interestingly, FITC-FNP-PEI exhibited a higher remaining percentage (69%) than FITC-FNP-PEG (39%), and FITC-FNP (29%) after 6 h continuous fluid flow. The nature of the polymer coating on the particles could be attributed as the primary factors influencing mucoadhesion. Several studied reported that the mucoadhesive properties of the nanoparticles could be enhanced by coating the particles with hydrophilic or cationic polymers via the interaction between the polymer and mucin chain [44]. PEI could increase the mucoadhesion on the mucus membrane through ionic interaction between the positively charged PEI and the negatively charged mucin. On the other hand, PEG, being a hydrophilic polymer with abundant hydroxyl contents, facilitates the penetration of the polymer chain into the mucus layer and engages in hydrogen bonding with mucin [45]. Based on these results, FNP-PEI and FNP-PEG exhibited strong adhesion on the mucus membrane when compared with uncoated FNP. Moreover, mucoadhesive properties of FNPs and FNPs-ISG formulations were compared. As expected, the FITC-FNP-ISG (Figure 4b) and FITC-FNP-PEG-ISG (Figure 4d) exhibited significantly greater FITC remaining than their particles only. This result could be attributed to the increasing viscosity of the gelling system. Therefore, the combination of in situ gelling and nanoparticles could prolong the drug retention on the ocular surface. However, the FITC-FNP-PEI in situ hydrogel (Figure 4c) showed lower FITC remaining than FITC-FNP-PEI particles. This result might be due to the hydrogel network retarding the interaction between positively charged PEI and negatively charged mucin.

### 3.5. Ex Vivo Mucoadhesive Study

**Figure 5:**
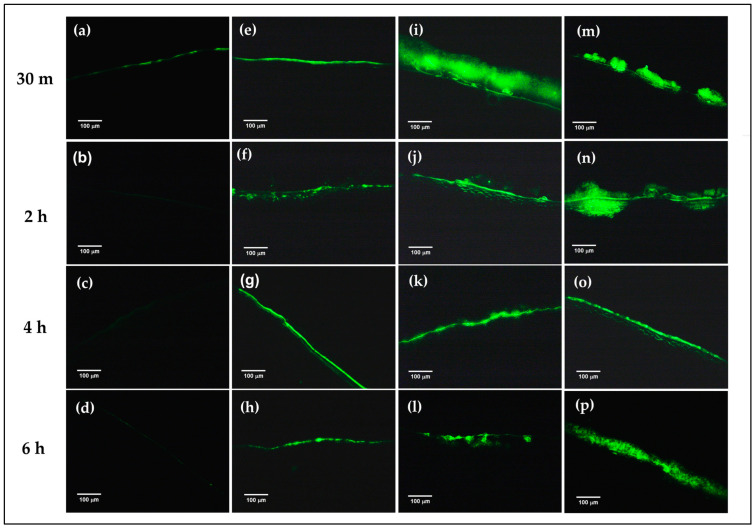
Ex vivo mucoadhesive studies of the nanoparticles and in situ hydrogel compared with nanodispersion and solution formulations. The fluorescence images of the remaining fluorescence on the cross-sectional porcine cornea after treatment with FITC solution (**a**–**d**), FITC-labeled FNP-PEG (**e**–**h**), FNP-PEG-F127 ISG (**i**–**l**), and FNP-PEG-F127/HA ISG (**m**–**p**) under continuous flow of STF at different time points (200×).

To confirm the mucoadhesion of the prepared formulations on the corneal surface, FITC-FNP-PEG in situ hydrogel and FITC-FNP-PEG were chosen for ex vivo mucoadhesive study. Similar to the in vitro mucoadhesive results, the FITC-FNP-PEG (Figure 5e–h), FITC-FNP-PEG-F127 ISG (Figure 5i–l), and FITC-FNP-PEG-F127/HA ISG (Figure 5m–p) showed good adhesion to the porcine cornea up to 6 h, while the FITC solution (Figure 5a–d) exhibited low intensity of green fluorescence at 30 min and was completely cleared away at 2 h. As expected, FITC-FNP-PEG-F127 in situ hydrogels showed higher intensity of green fluorescence for all time points than FITC-FNP-PEG under fluid flow. These results confirmed that the combination of FNP and in situ hydrogels could enhance the adhesion ability of the particles on the ocular surface. 

### 3.6. In Vitro Eye Irritation Studies

During drug development, the ocular irritation potential and toxicity of the ocular formulation must be tested to ensure the safety and biocompatibility of the product before clinical trial in humans. The in vitro cell model is one of the most applicable to eye irritation assessment because it is inexpensive, simple, and quick to implement compared to in vivo testing. In this study, the cytotoxicity study was carried out using the HCE cell line and modified following the STE protocol, as suggested for the assessment of eye irritation potential rather than animal testing [34,46]. The toxicity was accessed using MTT reagent based on mitochondrial activity, which is proportional to the number of viable cells, and the sample demonstrating cell viability higher than 70% was classified as a non-irritant [34]. 

**Figure 6:**
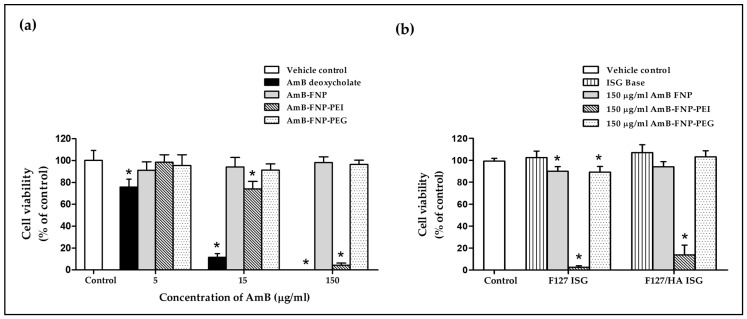
In vitro cytotoxicity study of prepared formulations. (**a**) Percentage of HCE cell viability after 12 h incubation with AmB deoxycholate and AmB-FNP formulations (equivalent concentration of AmB at 5, 15, and 150 μg/mL). (**b**) Percentage of HCE cell viability after 12 h incubation with AmB-FNPs embedded in F127 and F127/HA in situ hydrogel (formulation dose at 150 μg/mL of AmB). (mean ± SD, *n* = 9, * *p* < 0.05).

The effect of AmB-loaded FNPs on HCE cell viability is shown in Figure 6a. The exposure of HCE cells to AmB deoxycholate containing 5, 15, and 150 μg/mL of AmB exhibited cell viability of 76%, 12%, and 0% respectively, demonstrating their severe irritation effect. These toxic effects were associated with the aggregation state of AmB in this formulation, which was confirmed by the absorption spectra from our previous report [27]. Several studies have reported that the aggregated AmB form could bind to both ergosterol in fungal cells and cholesterol in mammalian cells, resulting in leakage of metabolites and ions from the cell membrane, and eventually leading to cell death [47]. In addition, sodium deoxycholate acted as a surfactant in this formulation, which could enhance cell membrane permeability of both mammalian and fungal cells, leading to cell damage [48,49]. 

Interestingly, all concentrations of AmB-FNP and AmB-FNP-PEG exhibited cell viability > 90%, and could therefore be categorized as a non-irritant and safe for the eye. The lower toxicity of both formulations could be explained by the AmB encapsulated in silk fibroin nanoparticles, which could reduce molecular aggregation of AmB, as characterized by the absorbance ratio of the first to fourth peaks from UV–Vis spectroscopy, indicating a higher specificity to ergosterol than cholesterol [27]. Moreover, AmB-FNP and AmB-FNP-PEG, which are composed of fibroin and PEG 400, are biocompatible with ocular tissue; consequently, these formulations exhibited cell viability similar to that of the control and greater than that of AmB deoxycholate.

Unfortunately, AmB-FNP-PEI showed a toxic effect to HCE cells at the high concentration even though this formulation exhibited the partial aggregation of AmB. The exposure of HCE cells to AmB-FNP-PEI containing 5, 15, and 150 μg/mL of AmB possessed cell viability of 98%, 74%, and 4%, respectively, which indicated classification as a potential irritant at the high dose. This toxic effect was related to the amount of PEI because the blank FNP-PEI also showed the toxicity to HCE cells in a dose-dependent manner (data not shown). Although PEI is capable of binding with the negative charge of mucin on the ocular surface, which enhances the precorneal retention time, it is also toxic like other cationic polymers. Fischer et al. reported that the high charge density of branching PEI can interact with the negative charge of the cell membrane, leading to weakening of the plasma membrane integrity and causing cell death [50]. In addition, they also found that the PEI affected the metabolic activity, and the severity of cytotoxic effects depends on the exposure time and concentration of the polymer. 

Figure 6b demonstrates the cell viability of the HCE cells after exposure to ISG bases, AmB-FNPs F127 ISG, and AmB-FNPs F127/HA ISG. Both ISG bases showed % cell viability having no significant difference from the control, and thus indicating no irritation. The HCE cells exposed to AmB-FNP-ISG and AmB-FNP-PEG ISG showed viability of more than 80%, suggesting no irritation and that they are safe for ocular uses. However, the exposure of HCE cells to AmB-FNP-PEI ISG exhibited cell viability of less than 20%, indicating a toxic effect due to PEI as described above.

**Figure 7:**
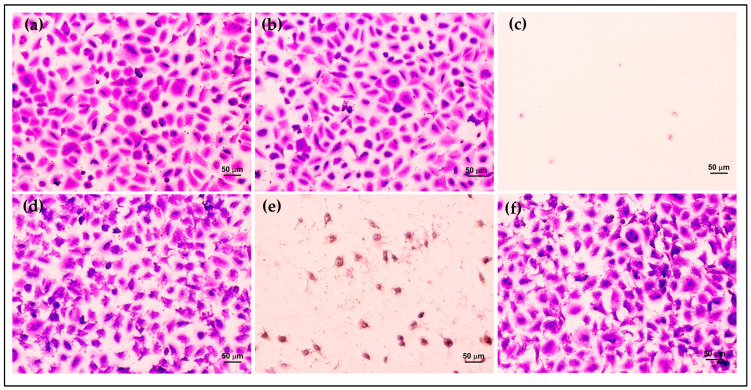
Morphology of HCE cells after exposure to AmB-FNP dispersions compared with the controls for 12 h: (**a**) untreated cells; (**b**) vehicle control; (**c**) 150 μg/mL AmB deoxycholate; (**d**) 150 μg/mL AmB-FNP; (**e**) 150 μg/mL AmB-FNP-PEI; and (**f**) 150 μg/mL AmB-FNP-PEG (200×).

**Figure 8:**
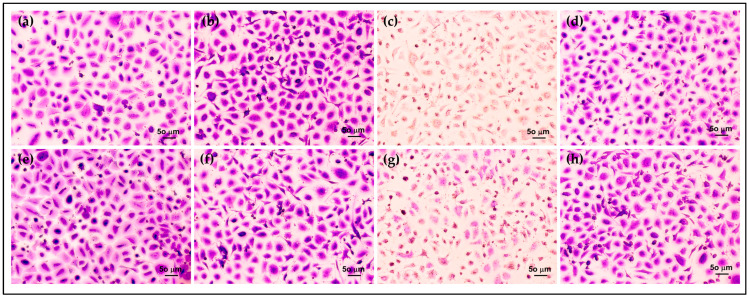
Morphology of HCE cells after exposure to AmB-FNPs ISG compared with their hydrogel bases for 12 h: (**a**) F127 ISG base; (**b**) AmB-FNP-F127; (**c**) AmB-FNP-PEI F127; (**d**) AmB-FNP-PEG F127; (**e**) F127/HA ISG base; (**f**) AmB-FNP-F127/HA; (**g**) AmB-FNP-PEI-F127/HA; and (**h**) AmB-FNP-PEG-F127/HA (200×).

Furthermore, the morphology and density of HCE cells after treatment were assessed via crystal violet staining. The morphology of HCE cells treated with AmB-FNPs and AmB-FNP-ISG are shown in Figure 7 and Figure 8, respectively. When compared with untreated cells and the vehicle control, the cells exposed with AmB-FNP, AmB-FNP-PEG, and their AmB-FNP in situ hydrogels revealed no differences in cell number and morphology from the control cells. These results confirmed that AmB-FNP-ISG and AmB-FNP-PEG-ISG have good biocompatibility and are safe for topical ocular application. In contrast, the cells exposed to AmB deoxycholate, which is the marketed formulation, demonstrated a small amount of cell debris remaining on the well plate, indicating significant cell death and detachment from the surface. Additionally, the cell exposure to AmB-FNP-PEI and its hydrogel revealed cell debris and changes in morphology, indicating cell damage and unsuitability for ocular application.

## 4. Conclusions

Our current study demonstrates the efficacy and safety of the combination system of AmB-FNPs-ISG as mucoadhesive ophthalmic eye drops for FK treatment. The optimized AmB-FNPs demonstrated a spherical shape with a mean particle size of 215 nm and high entrapment efficiency of 71%. The developed thermosensitive AmB-FNP in situ hydrogel formulations displayed satisfactory gelling capacity, a translucent homogeneous solution, pH ~7, osmolality of ~323–348 mOsmol/kg, and %transmittance > 90%. All composite formulations illustrated optimal viscosity and pseudoplastic behavior, which are suitable for ocular application. Furthermore, the nanoparticle in situ hydrogel formulations showed good physiochemical stability under storage conditions for 90 days. The combined systems of AmB-FNPs-ISG exhibited an effective antifungal effect against *C. albicans* similar to that of commercial AmB, and they showed a greater antifungal effect than the single AmB-FNP as a result of the synergistic effect of the Pluronic surfactant in the hydrogel. As expected, in vitro and ex vivo mucoadhesive results of the combined system showed higher fluorescence intensity than the solution and nanodispersion, which confirmed that the combination of FNPs with in situ hydrogels could enhance the retention time of the particles on the corneal tissue for more than 6 h. However, AmB-FNP-PEI ISG demonstrated toxicity to the HCE cells depending on the PEI content in the particles, whereas AmB-FNP-ISG, AmB-FNP-PEG-ISG, and their FNP counterparts exhibited significantly less toxicity on HCE cells than commercial AmB, thus making them more suitable for ocular application. Thus, the smart AmB-FNP-PEG-ISG has potential as ready-to-use AmB eye drops for FK treatment.

## Figures and Tables

**Table 1:** Physicochemical characterization of AmB-FNPs and AmB-FNPs-ISG composites.

Parameters	AmB-FNP-ISG		AmB-FNP-PEI-ISG		AmB-FNP-PEG-ISG	
	F127	F127/HA	F127	F127/HA	F127	F127/HA
Particle size, shape	206.8 ± 5.6 nm, spherical		209.0 ± 14.4 nm, cubic		214.7 ± 15.9 nm, spherical	
Zeta potential	−23.13 ± 2.69 mV		35.87 ± 1.37 mV		−22.04 ± 1.81 mV	
PDI	0.11 ± 0.05		0.18 ± 0.03		0.15 ± 0.01	
EE/DL	63.2%/8.7%		72.6%/5.2%		71.3%/9.7%	
Gelling capacity	++	++	++	++	++	++
pH	7.2 ± 0.2	6.7 ± 0.1	6.9 ± 0.1	6.7 ± 0.1	6.9 ± 0.1	6.9 ± 0.1
Osmolality (mOsm/kg)	338 ± 12	328 ± 9	348 ± 14	347 ± 11	332 ± 8	323 ± 7
%T (381–780 nm)	98 ± 5	97 ± 6	97 ± 5	96 ± 6	98 ± 4	97 ± 4
Viscosity (mPa·s)						
at 25 ± 1 °C	104 ± 2	324 ± 43	125 ± 31	280 ± 16	101 ± 12	315 ± 40
at 35 ± 1 °C	8214 ± 256	2706 ± 1349	7329 ± 1557	4140 ± 916	8233 ± 325	3571 ± 984

Note: PDI = polydispersity index, EE = entrapment efficiency, DL = drug loading capacity, gelling capacity: ++ = sol–gel transition within 30 s, and %T = % transmittance.

**Table 2:** Minimum inhibitory concentration (MIC90) and minimum fungicidal concentration (MFC) of the formulations against *C. albicans* (*n* = 3).

Formulation	MIC_90_ (μg/mL)	MFC (μg/mL)
AmB Deoxycholate	0.0625	0.5
Standard AmB	0.0625	0.5
AmB-FNP	0.250	1
AmB-FNP-PEI	0.250	2
AmB-FNP-PEG	0.250	1
AmB-FNP-F127 ISG	0.125	0.5
AmB-FNP-PEI-F127 ISG	0.125	0.5
AmB-FNP-PEG-F127 ISG	0.125	0.5
AmB-FNP-F127/HA ISG	0.125	0.5
AmB-FNP-PEI-F127/HA ISG	0.125	0.5
AmB-FNP-PEG-F127/HA ISG	0.125	0.5

## Data Availability

The data presented in this study are available on request from the corresponding author.
